# In silico segmentations of lentivirus envelope sequences

**DOI:** 10.1186/1471-2105-8-99

**Published:** 2007-03-21

**Authors:** Aurélia Boissin-Quillon, Didier Piau, Caroline Leroux

**Affiliations:** 1 UMR754 INRA-ENVL-UCBL "Rétrovirus et Pathologie Comparée", IFR 128 BioSciences Lyon-Gerland, Université Claude Bernard Lyon 1, 69007 Lyon, France; 2 Institut Fourier UMR 5582 CNRS-UJF, Université Joseph Fourier (Grenoble 1), 100 rue des Maths, BP 74, 38402 Saint Martin d'Hères, France

## Abstract

**Background:**

The gene encoding the envelope of lentiviruses exhibits a considerable plasticity, particularly the region which encodes the surface (SU) glycoprotein. Interestingly, mutations do not appear uniformly along the sequence of SU, but they are clustered in restricted areas, called variable (V) regions, which are interspersed with relatively more stable regions, called constant (C) regions. We look for specific signatures of C/V regions, using hidden Markov models constructed with SU sequences of the equine, human, small ruminant and simian lentiviruses.

**Results:**

Our models yield clear and accurate delimitations of the C/V regions, when the test set and the training set were made up of sequences of the same lentivirus, but also when they were made up of sequences of different lentiviruses. Interestingly, the models predicted the different regions of lentiviruses such as the bovine and feline lentiviruses, not used in the training set. Models based on composite training sets produce accurate segmentations of sequences of all these lentiviruses.

**Conclusion:**

Our results suggest that each C/V region has a specific statistical oligonucleotide composition, and that the C (respectively V) regions of one of these lentiviruses are statistically more similar to the C (respectively V) regions of the other lentiviruses, than to the V (respectively C) regions of the same lentivirus.

## Background

Retroviruses are RNA viruses infecting vertebrates and many non vertebrates. Virus particles are spherical and surrounded by an envelope. Their viral replication is dependent of the RT (Reverse Transcriptase), a viral RNA-dependent DNA-polymerase. The lentivirus genus is part of the retrovirus family. Lentiviruses infect animals and humans and cause slowly progressing diseases. Among the lentivirus genus, HIV-1 and HIV-2 (Human Immunodeficiency Virus type 1 and 2) infect humans, EIAV (Equine Infectious Anemia Virus) infects equids, SRLV (Small Ruminant LentiVirus) infects goats and sheep, SIV (Simian Immunodeficiency Virus) infects non primate monkeys, BIV (Bovine Immunodeficiency Virus) infects bovines and FIV (Feline Immunodeficiency Virus) infects felines.

The considerable plasticity of the genome of lentiviruses is quite obvious in the *env *gene, encoding the envelope, particularly in the region encoding the surface (SU) glycoprotein forming spikes. Causes of this plasticity are, among other factors, the low fidelity of the viral reverse transcriptase (RT) during the retrotranscription of the viral RNA genome into DNA, the lack of proofreading activity of the RT, the high level of virus replication, and some recombination events in co-infected cells [1-4].

Interestingly, SU mutations do not appear uniformly along the *env *gene, but are clustered in restricted and specific areas defined as variable (V) regions flanked by constant (C) regions. On average, and depending on the lentivirus considered, from 10 % to 35 % of the amino-acids in SU vary between isolates, and more than 70 % of these variable amino-acids are located in V regions. Such C/V segmentations hold for all the lentiviruses [5-11].

It is unclear whether the accumulation of mutations in V regions is mainly due to locally high intrinsic mutation rates, or if mutations occur at similar rates at every SU sites with subsequent selection mechanisms eliminating most variants from the C regions. In any case, the plasticity of these genomes allows them to escape immune control very efficiently, while keeping their identity. Most of amino acids encoded by the V regions are on the outside of SU, while the amino acids encoded by the C regions are in the internal part. In this respect, one should note that the replication acts on one-dimensional molecules, at a moment when most of the information about their three-dimensional conformation seems unavailable. In other words, if the intrinsic mutation rates are indeed different in C regions and in V regions, this might be due to some specific signals encoded by the nucleotide (linear) viral sequence itself, possibly corresponding to interactions with the RT. To test this hypothesis, we developed a mathematical model based on lentivirus sequences, as simple and robust as possible, able to localize and to characterize their C/V segmentation of the SU region. Our approach was based on HMMs (hidden Markov models). These models are tailored to describe heterogeneous sequences, since they basically break down a given sequence into a succession of locally homogeneous subsequences. HMMs were initially introduced in the context of speech recognition [12] and they are now major tools of the analysis of genomic and proteomic sequences [13-19]. In sequence analysis, each of the subsequences called a region, is described by the value of a Markov chain, called the hidden state, taken from a finite collection of values. Each state is characterized by its own statistical composition in nucleotides or in amino-acids. The succession of states itself is ruled by a master Markov chain, called the hidden chain.

Our main findings are as follows. Using SU sequences of EIAV, HIV, SRLV or SIV to train the HMMs, we obtained clear and accurate delimitations of the C and V regions of these lentiviruses. This suggests that the statistical composition of the C regions is markedly different from the statistical composition of the V regions. Additionally, we developed combined models, based on EIAV, HIV, SIV and SRLV sequences. These were able to predict simultaneously the C and V regions of every lentivirus in the collection above. Our combined models also predicted the C/V segmentation of other lentiviruses which were not used in the training sets: BIV and FIV. This indicates that the C and V regions are statistically distinct and that the V regions of all the lentiviruses share common statistical signatures.

## Results

### C/V segmentations of EIAV

We first tried to differentiate the C and V regions of the EIAV SU, using HMMs with *N *= 2 hidden states, for different orders *m*. The parameters of the models were estimated on training sets of 94 nucleotide sequences, by the EM algorithm. We used various training sets, dividing at random our complete set of sequences into two equal parts (1:1), the training and the test sets. Then, none of the various HMMs was able to identify the C and V regions of the EIAV SU. We obtained hidden states sequences which oscillated and repeatedly jumped from one hidden state to the other (data not shown). Hence, this method was not reliable to identify homogeneous regions corresponding to the C and V regions of the EIAV SU.

By contrast, fixed EM, as described in section Methods, yielded a clear delimitation of the known C and V regions on the whole test set, for HMMs of different order. For example, HMM of order *m *= 2 allowed us to predict most of the V regions on nucleotide sequences. HMMs of higher orders (*m *≥ 3) gave even more accurate prediction. To evaluate the fit quality and to select the best model among the candidates, we used the Akaike information criterion (AIC) [20]. This criterion is defined by

**Figure 1:**
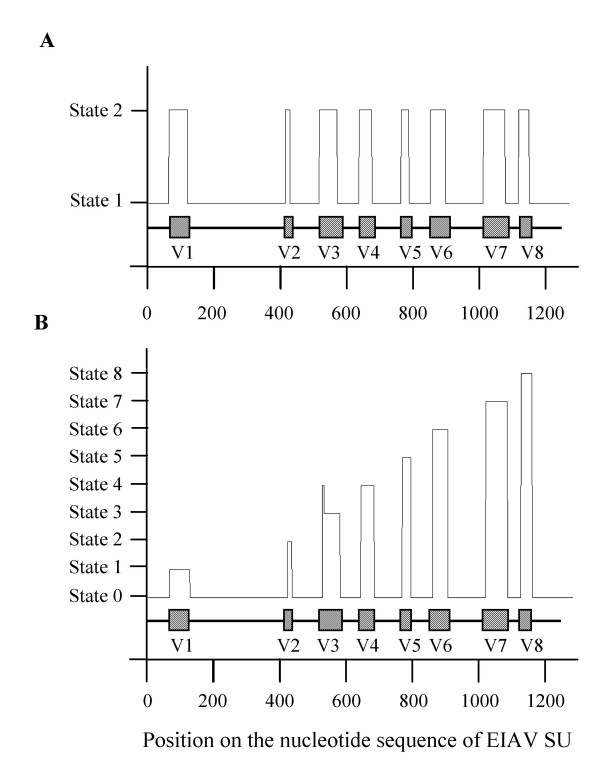
**Regions predicted by the hidden Markov models on the nucleotide sequence of EIAV SU**. The graphic displays the regions predicted by our mathematical model (—). The schematic organization of EIAV SU, with the position of the 8 variable regions V1 to V8 (hatched boxes) and of the 9 constant regions (—), as defined by classical amino-acid multiple alignments, is represented under the graphic. (A) HMM of order 5 with 2 hidden states, trained on the variable (V) and constant (C) regions. (B) HMM of order 5 with 9 hidden states, trained on the 8 V regions and on the reunion of the C regions.

*AIC *= -2log(*L*) + 2*n*,

where *L *is the likelihood of the candidate model and *n *the number of free parameters. AIC is based on the Kullback-Leibler distance between different distributions of sequences (for this notion, see our section below on Separation of the EIAV C/V regions) and is designed to achieve a balance between fit quality and number of parameters corresponding to the model with the lowest AIC. The best model to predict the C and V regions on nucleotide sequences of the EIAV SU was of order *m *= 6, according to the AIC criterion (Table 1). However, for *m *= 5, the fit of the predicted C and V regions with the segmentations deduced from alignments was almost perfect (Figure 1A) and minimized the risk of overfitting the data.

**Table 1:** Modeling of the C and V regions of the EIAV SU.

Number of hidden states			
N = 2		N = 9	
Order	AIC	Order	AIC

m = 2	250184	m = 2	240054
m = 3	233586	m = 3	215632
m = 4	189932	m = 4	180734
m = 5	138084	**m = 5**	**169786**
**m = 6**	**130774**	m = 6	300404
m = 7	262786		

The best model according to AIC (Akaike Information Criterion), i.e. the one with the smallest AIC value, is shown in bold.

To differentiate the variable regions V1 to V8 and the C regions, we then used HMMs with *N *= 9 states on nucleotide sequences. Thus, we trained one hidden state with each variable region and one hidden state with the constant regions as a whole, and we estimated the parameters of a HMM of different orders by the fixed EM algorithm. The best model is of order *m *= 5 (Table 1). This yields a precise delimitation of the C and V regions on nucleotide sequences, each V region showing a distinct signal (Figure 1B). Estimating the parameters of the models with the direct counting methods gave similar results.

**Figure 2:**
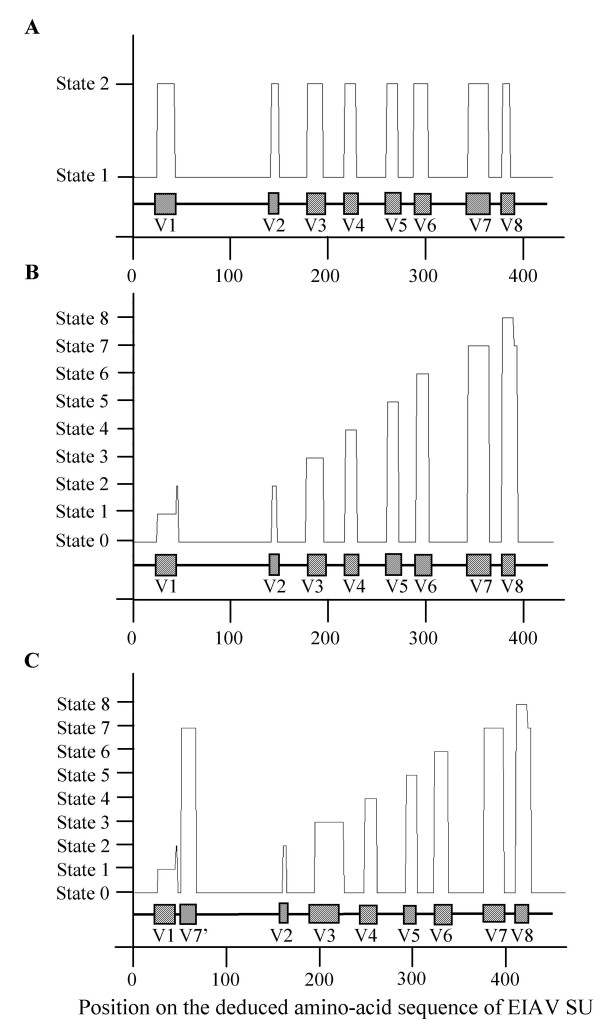
**Regions predicted by the hidden Markov models on the deduced amino-acid sequence of EIAV SU**. The graphic displays the regions predicted by our mathematical model (—). The schematic organization of EIAV SU with the position of the 8 variable regions V1 to V8 (hatched boxes) and of the 9 constant regions (—), as defined by classical amino-acid multiple alignments, is represented under the graphic. (A) First order HMM with 2 hidden states, trained on the V and C regions. (B) First order HMM with 9 hidden states, trained on the 8 V regions and on the reunion of the C regions. (C) First order HMM with 9 hidden states tested on an artificial sequence, where 15 amino-acids of the V7 sequence are inserted into the constant region C2 located between V1 and V2.

Finally, HMMs with *N *= 2 or *N *= 9 hidden states, able to locate the C and V regions on deduced amino-acid sequences, were trained by the fixed EM algorithm and the direct counting method. We obtained accurate predictions of the C and V regions on the test sequences, with every training method, using a HMM of order *m *= 1 (Figures 2A and 2B).

The reconstructed sequences of the hidden states did not oscillate between the different hidden states as in the models based on the EM algorithm. The transition matrix obtained without prior information on the length of the regions allowed to identify long homogeneous regions and to compare them to the C and V regions previously defined.

At this point, we developed models with an unique C region. This C region do not fit a real region but represent an average of all the constant regions. There is no guarantee *a priori *that the constant regions are grouped together and can be modeled by an unique state. However, the C region introduced in our models allowed to predict all the constant regions with an amazing accuracy.

### Tests of the models of EIAV C/V regions

Since our models were able to predict the C and V regions on both deduced amino-acid and nucleotide sequences of EIAV SU, we put them under trial in several directions. First, we checked that the models were not overfitted, keeping in mind that pseudo-counts were introduced to limit the overfitting problem. We checked whether the models were not overly specific of the training data, and whether it was possible to make them encompass new data tests. To perform such tests, the models were trained using nucleotide or amino-acid sequences sharing a minimal amount of motifs with the test sequences. For example, we trained the models on virus sequences, which were present at the beginning of the disease induced in horses by EIAV, and we tested them on virus sequences at later stages of the disease [6]. Because of the variations due to viral replication during the time course of the EIAV infection, the training and test sequences displayed 7.8 % (± 1.3) of divergence at the amino-acid level. In particular, the test and training sequences displayed 43.8 % (± 20.2) of divergence in the third V region (V3). Despite this important level of divergence between the training and test sequences, the models correctly predicted the C and V regions, notably V3.

To check that the models were not simply following the order and positions of the V regions along the sequence, we also assembled artificial sequences with a greater number of V regions than in the real ones. For instance, we inserted a copy of 15 amino-acids, taken from V7, into C2. The models which were trained with the fixed EM algorithm on the original sequences, managed to predict perfectly the additional V region of these modified sequences (Figure 2C).

### Combined C/V models

**Figure 3:**
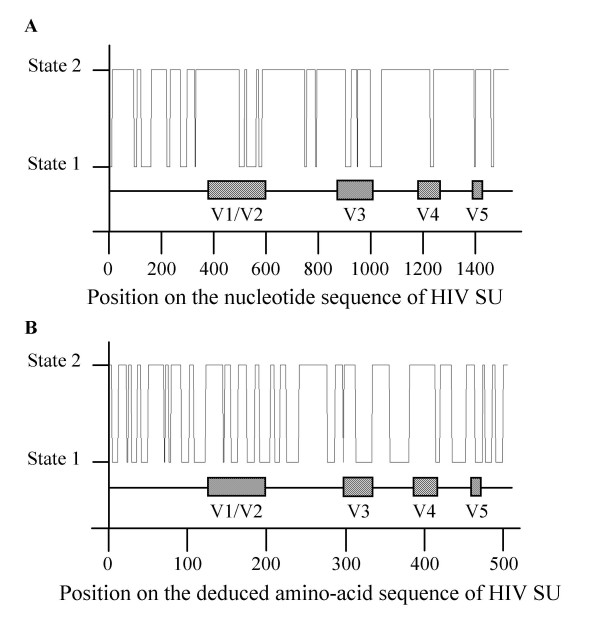
**Regions predicted on HIV sequences by hidden Markov models trained on EIAV sequences**. The graphic displays the regions predicted on the HIV-1 HXB2 sequence by our mathematical models (—). The schematic organization of HIV SU with the position of the variable regions (hatched boxes) and of the constant regions (—), as defined by classical amino-acid alignments, is represented under the graphic. (A) HMM of order 5 with 2 hidden states trained on nucleotide sequences of EIAV SU. (B) First order HMM with 2 hidden states trained on deduced amino-acid sequences of EIAV SU.

**Figure 4:**
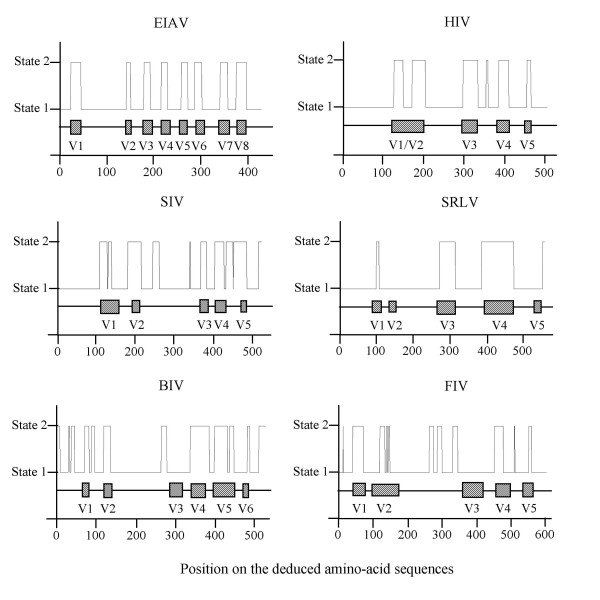
**Regions predicted by the combined hidden Markov model trained on EIAV, HIV, SIV, and SRLV SU**. The graphs display the regions predicted by the first order combined HMM, on sequences of EIAV, HIV, SIV, SRLV, BIV and FIV SU. The schematic organization of SU with the position of the variable regions (hatched boxes) and of the constant regions (—), as defined by classical amino-acid alignments, is represented under the graphics.

Models based on EIAV sequences were unable to predict C and V regions of HIV, SIV or SRLV SU sequences (Figure 3). Hence, we developed a new specific model for each lentivirus. We trained models of order *m *= 1 on deduced amino-acid sequences and models of order *m *= 5 on nucleotide sequences, on 78 HIV sequences, 45 SIV sequences and 51 SRLV sequences respectively, using either the fixed EM algorithm or the direct counting methods. These new models, specific to each lentivirus, were indeed able to predict the C and V regions of test sequences of the corresponding virus, but failed to predict the C and V regions of the other lentiviruses. On the contrary, a combined HMM of order *m *= 1 with *N *= 2 hidden states, trained on a composite training set of EIAV, HIV, SIV and SRLV deduced amino-acid sequences, was powerful enough to localize accurately the V regions of test sequences of EIAV (V1 to V8), HIV (V1 to V4), and SIV or SRLV (V1 to V5). Rather to our surprise, the model also discriminated V1 and V2 of HIV, although these two regions were given as a unique region V1/V2 in the training set (Figure 4). The C and V regions of EIAV, HIV, SIV and SRLV were also predicted with great accuracy by HMMs of order *m *= 5 with *N *= 2 hidden states, trained on the corresponding nucleotide sequences. Finally, the combined models, trained on EIAV, HIV, SIV and SRLV sequences, were able to predict C and V regions of two lentiviruses which were not used to train them, namely BIV and FIV (Figure 4).

### Separation of the EIAV C/V regions

The models developed in our study allow us to differentiate the C and V regions of EIAV and to distinguish each of the 8 variable regions. This indicates that the C and V regions have distinct statistical composition and that the 8 variable regions are statistically distinct too. A classical method to quantify the differences between the Markov chains which represent the C and V regions of EIAV, is to consider the relative entropy, also named Kullback-Leibler divergence, between these models, see [21-26]. The relative entropy of two Markov chains is given by

H(P|Q)=∑i,jπ(i)P(i,j)log⁡P(i,j)Q(i,j),
 MathType@MTEF@5@5@+=feaafiart1ev1aaatCvAUfKttLearuWrP9MDH5MBPbIqV92AaeXatLxBI9gBaebbnrfifHhDYfgasaacH8akY=wiFfYdH8Gipec8Eeeu0xXdbba9frFj0=OqFfea0dXdd9vqai=hGuQ8kuc9pgc9s8qqaq=dirpe0xb9q8qiLsFr0=vr0=vr0dc8meaabaqaciaacaGaaeqabaqabeGadaaakeaacqWGibascqGGOaakcqWGqbaucqGG8baFcqWGrbqucqGGPaqkcqGH9aqpdaaeqbqaaGGaciab=b8aWjabcIcaOiabdMgaPjabcMcaPiabdcfaqjabcIcaOiabdMgaPjabcYcaSiabdQgaQjabcMcaPiGbcYgaSjabc+gaVjabcEgaNnaalaaabaGaemiuaaLaeiikaGIaemyAaKMaeiilaWIaemOAaOMaeiykaKcabaGaemyuaeLaeiikaGIaemyAaKMaeiilaWIaemOAaOMaeiykaKcaaaWcbaGaemyAaKMaeiilaWIaemOAaOgabeqdcqGHris5aOGaeiilaWcaaa@5750@

where *P *and *Q *are the transition matrix of the two Markov chains and *π *the invariant distribution associated to *P*. We used a symmetrized form of the relative entropy, defined as

*δ*(*P*,*Q*) = *H*(*P*|*Q*) + *H*(*Q*|*P*).

The computation of the symmetrized relative entropy between the Markov chains modeling the 9 constant regions and the 8 variable regions of EIAV (see Table 2) indicates that the different C (respectively V) regions are closer to the global C (respectively V) region than to any of the V (respectively C) regions. Furthermore, the V regions are closer to each other than to any of the C regions.

**Table 2:** Symmetrized relative entropy between the C/V regions of EIAV.

*δ*	C1	C2	C3	C4	C5	C6	C7	C8	C9	C	V	V1	V2	V3	V4	V5	V6	V7	V8
C1	0	4.40	5.08	4.63	4.65	5.08	4.26	4.70	3.52	2.55	3.70	3.62	2.80	3.67	3.77	3.37	3.70	4.00	3.25
C2		0	6.25	5.77	5.93	6.38	5.90	4.65	5.37	2.58	4.60	4.46	3.47	4.53	4.72	4.96	4.94	4.72	3.78
C3			0	6.00	6.62	5.69	5.77	5.35	5.35	2.85	4.86	4.78	4.07	4.51	4.71	4.87	5.18	4.76	4.73
C4				0	6.36	5.71	5.22	5.06	5.09	2.65	4.95	4.70	3.48	4.85	5.14	5.04	4.13	5.26	4.28
C5					0	6.86	6.08	6.17	5.00	2.97	4.88	4.81	3.64	4.96	5.35	4.95	5.41	4.71	4.54
C6						0	6.21	4.70	5.25	3.18	4.70	4.60	3.40	4.75	4.74	4.75	4.67	5.20	3.90
C7							0	4.92	4.85	2.37	4.71	5.15	3.93	5.16	4.99	4.93	4.63	4.47	4.88
C8								0	4.34	2.66	4.68	3.77	3.20	4.25	4.11	4.80	4.04	4.50	3.36
C9									0	2.48	3.84	3.92	3.22	4.16	4.32	4.13	4.84	4.00	3.32
C										0	3.35	3.64	3.19	3.70	3.78	3.62	3.99	2.96	3.01
V											0	2.25	2.07	1.91	2.11	2.09	2.21	1.87	2.36
V1												0	2.41	3.79	3.26	3.07	3.59	3.64	3.57
V2													0	2.79	2.73	2.71	2.59	2.77	2.34
V3														0	3.63	3.77	3.60	4.54	3.41
V4															0	3.57	3.66	4.09	4.03
V5																0	4.08	3.77	3.69
V6																	0	4.25	3.52
V7																		0	3.83
V8																			0

**Figure 5:**
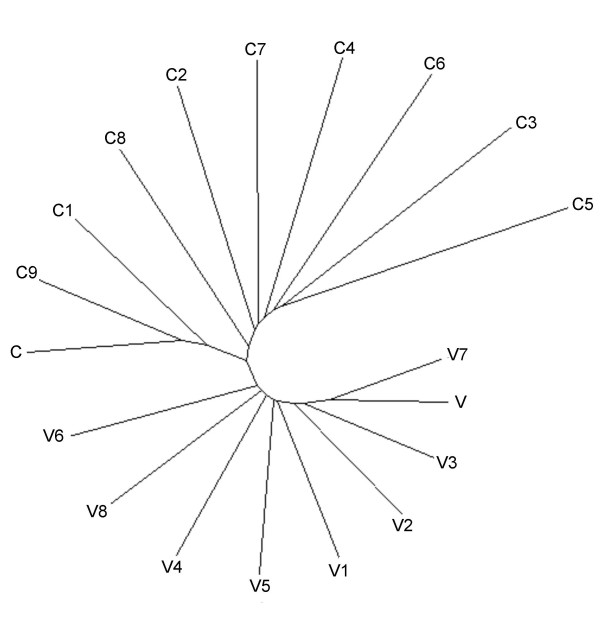
**Graphic representation of the distances between the C and V regions of EIAV**. A distance matrix between the C and V regions is computed with the symmetrised form of the relative entropy. A dendogram was evaluated with the Kitsch (Phylip 3.5c) program with the default parameters and drawn with the "Unrooted" software [41].

To quantify this overall feeling, we first used the symmetrized relative entropy *δ *to study the distances between the C and V regions, representing them by a dendogram. Note that *δ *is not a true metric because it does not satisfy the triangle inequality. However, one can visualize the distances between the different regions by an unrooted tree, computed by the program Kitch (Phylip 3.5c) using the distance matrix previously estimated (Figure 5). The dendogram shows a distinct separation between a first group, made of the C regions, and a second group, made of the V regions. This confirms the fact that the C and V regions of EIAV differ in their statistical composition.

To further quantify this separation between the C and V regions, we built an asymptotic statistical test for the empirical transition matrices of two different regions, based on the following considerations. Assuming in general that q1^
 MathType@MTEF@5@5@+=feaafiart1ev1aaatCvAUfKttLearuWrP9MDH5MBPbIqV92AaeXatLxBI9gBaebbnrfifHhDYfgasaacH8akY=wiFfYdH8Gipec8Eeeu0xXdbba9frFj0=OqFfea0dXdd9vqai=hGuQ8kuc9pgc9s8qqaq=dirpe0xb9q8qiLsFr0=vr0=vr0dc8meaabaqaciaacaGaaeqabaqabeGadaaakeaadaqiaaqaaiabdghaXnaaBaaaleaacqaIXaqmaeqaaaGccaGLcmaaaaa@2FFF@ and q2^
 MathType@MTEF@5@5@+=feaafiart1ev1aaatCvAUfKttLearuWrP9MDH5MBPbIqV92AaeXatLxBI9gBaebbnrfifHhDYfgasaacH8akY=wiFfYdH8Gipec8Eeeu0xXdbba9frFj0=OqFfea0dXdd9vqai=hGuQ8kuc9pgc9s8qqaq=dirpe0xb9q8qiLsFr0=vr0=vr0dc8meaabaqaciaacaGaaeqabaqabeGadaaakeaadaqiaaqaaiabdghaXnaaBaaaleaacqaIYaGmaeqaaaGccaGLcmaaaaa@3001@ are empirical transition matrices of the same Markov chain with theoretical transition matrix *q*, based on two independent sequences of length *L *of the Markov chain, one can show that *LH*(q1^
 MathType@MTEF@5@5@+=feaafiart1ev1aaatCvAUfKttLearuWrP9MDH5MBPbIqV92AaeXatLxBI9gBaebbnrfifHhDYfgasaacH8akY=wiFfYdH8Gipec8Eeeu0xXdbba9frFj0=OqFfea0dXdd9vqai=hGuQ8kuc9pgc9s8qqaq=dirpe0xb9q8qiLsFr0=vr0=vr0dc8meaabaqaciaacaGaaeqabaqabeGadaaakeaadaqiaaqaaiabdghaXnaaBaaaleaacqaIXaqmaeqaaaGccaGLcmaaaaa@2FFF@, q2^
 MathType@MTEF@5@5@+=feaafiart1ev1aaatCvAUfKttLearuWrP9MDH5MBPbIqV92AaeXatLxBI9gBaebbnrfifHhDYfgasaacH8akY=wiFfYdH8Gipec8Eeeu0xXdbba9frFj0=OqFfea0dXdd9vqai=hGuQ8kuc9pgc9s8qqaq=dirpe0xb9q8qiLsFr0=vr0=vr0dc8meaabaqaciaacaGaaeqabaqabeGadaaakeaadaqiaaqaaiabdghaXnaaBaaaleaacqaIYaGmaeqaaaGccaGLcmaaaaa@3001@) is asymptotically *χ*^2^-distributed with *D*(*q*) degrees of freedom, where *D*(*q*) denotes the "dimension" of the Markov chain, that is, *D*(*q*) is the number of nonzero coefficients in *q *minus the number of states [see Additional file 1]. When every transition has positive probability and *q *has size *M*, *D*(*q*) = *M*^2 ^- *M*. In particular,

*E*(*H*(q1^
 MathType@MTEF@5@5@+=feaafiart1ev1aaatCvAUfKttLearuWrP9MDH5MBPbIqV92AaeXatLxBI9gBaebbnrfifHhDYfgasaacH8akY=wiFfYdH8Gipec8Eeeu0xXdbba9frFj0=OqFfea0dXdd9vqai=hGuQ8kuc9pgc9s8qqaq=dirpe0xb9q8qiLsFr0=vr0=vr0dc8meaabaqaciaacaGaaeqabaqabeGadaaakeaadaqiaaqaaiabdghaXnaaBaaaleaacqaIXaqmaeqaaaGccaGLcmaaaaa@2FFF@, q2^
 MathType@MTEF@5@5@+=feaafiart1ev1aaatCvAUfKttLearuWrP9MDH5MBPbIqV92AaeXatLxBI9gBaebbnrfifHhDYfgasaacH8akY=wiFfYdH8Gipec8Eeeu0xXdbba9frFj0=OqFfea0dXdd9vqai=hGuQ8kuc9pgc9s8qqaq=dirpe0xb9q8qiLsFr0=vr0=vr0dc8meaabaqaciaacaGaaeqabaqabeGadaaakeaadaqiaaqaaiabdghaXnaaBaaaleaacqaIYaGmaeqaaaGccaGLcmaaaaa@3001@)) ~ *D*(*q*)/*L*.

In the still more general case when q1^
 MathType@MTEF@5@5@+=feaafiart1ev1aaatCvAUfKttLearuWrP9MDH5MBPbIqV92AaeXatLxBI9gBaebbnrfifHhDYfgasaacH8akY=wiFfYdH8Gipec8Eeeu0xXdbba9frFj0=OqFfea0dXdd9vqai=hGuQ8kuc9pgc9s8qqaq=dirpe0xb9q8qiLsFr0=vr0=vr0dc8meaabaqaciaacaGaaeqabaqabeGadaaakeaadaqiaaqaaiabdghaXnaaBaaaleaacqaIXaqmaeqaaaGccaGLcmaaaaa@2FFF@ and q2^
 MathType@MTEF@5@5@+=feaafiart1ev1aaatCvAUfKttLearuWrP9MDH5MBPbIqV92AaeXatLxBI9gBaebbnrfifHhDYfgasaacH8akY=wiFfYdH8Gipec8Eeeu0xXdbba9frFj0=OqFfea0dXdd9vqai=hGuQ8kuc9pgc9s8qqaq=dirpe0xb9q8qiLsFr0=vr0=vr0dc8meaabaqaciaacaGaaeqabaqabeGadaaakeaadaqiaaqaaiabdghaXnaaBaaaleaacqaIYaGmaeqaaaGccaGLcmaaaaa@3001@ are based on independent sequences of unequal lengthes *L*_1 _and *L*_2 _respectively, a similar result holds, namely that ℓ *H*(q1^
 MathType@MTEF@5@5@+=feaafiart1ev1aaatCvAUfKttLearuWrP9MDH5MBPbIqV92AaeXatLxBI9gBaebbnrfifHhDYfgasaacH8akY=wiFfYdH8Gipec8Eeeu0xXdbba9frFj0=OqFfea0dXdd9vqai=hGuQ8kuc9pgc9s8qqaq=dirpe0xb9q8qiLsFr0=vr0=vr0dc8meaabaqaciaacaGaaeqabaqabeGadaaakeaadaqiaaqaaiabdghaXnaaBaaaleaacqaIXaqmaeqaaaGccaGLcmaaaaa@2FFF@, q2^
 MathType@MTEF@5@5@+=feaafiart1ev1aaatCvAUfKttLearuWrP9MDH5MBPbIqV92AaeXatLxBI9gBaebbnrfifHhDYfgasaacH8akY=wiFfYdH8Gipec8Eeeu0xXdbba9frFj0=OqFfea0dXdd9vqai=hGuQ8kuc9pgc9s8qqaq=dirpe0xb9q8qiLsFr0=vr0=vr0dc8meaabaqaciaacaGaaeqabaqabeGadaaakeaadaqiaaqaaiabdghaXnaaBaaaleaacqaIYaGmaeqaaaGccaGLcmaaaaa@3001@) is asymptotically *χ*^2^-distributed with *D*(*q*) degrees of freedom, where ℓ denotes the harmonic mean of *L*_1 _and *L*_2_, defined by the relation

2ℓ=1L1+1L2.
 MathType@MTEF@5@5@+=feaafiart1ev1aaatCvAUfKttLearuWrP9MDH5MBPbIqV92AaeXatLxBI9gBaebbnrfifHhDYfgasaacH8akY=wiFfYdH8Gipec8Eeeu0xXdbba9frFj0=OqFfea0dXdd9vqai=hGuQ8kuc9pgc9s8qqaq=dirpe0xb9q8qiLsFr0=vr0=vr0dc8meaabaqaciaacaGaaeqabaqabeGadaaakeaadaWcaaqaaiabikdaYaqaaiabloriSbaacqGH9aqpdaWcaaqaaiabigdaXaqaaiabdYeamnaaBaaaleaacqaIXaqmaeqaaaaakiabgUcaRmaalaaabaGaeGymaedabaGaemitaW0aaSbaaSqaaiabikdaYaqabaaaaOGaeiOla4caaa@383B@

Using the symmetrized entropy *δ*, one sees that the distribution of 12ℓδ(q1^,q2^)
 MathType@MTEF@5@5@+=feaafiart1ev1aaatCvAUfKttLearuWrP9MDH5MBPbIqV92AaeXatLxBI9gBaebbnrfifHhDYfgasaacH8akY=wiFfYdH8Gipec8Eeeu0xXdbba9frFj0=OqFfea0dXdd9vqai=hGuQ8kuc9pgc9s8qqaq=dirpe0xb9q8qiLsFr0=vr0=vr0dc8meaabaqaciaacaGaaeqabaqabeGadaaakeaadaWcaaqaaiabigdaXaqaaiabikdaYaaacqWItecBiiGacqWF0oazcqGGOaakdaqiaaqaaiabdghaXnaaBaaaleaacqaIXaqmaeqaaaGccaGLcmaacqGGSaaldaqiaaqaaiabdghaXnaaBaaaleaacqaIYaGmaeqaaaGccaGLcmaacqGGPaqkaaa@3AB5@ is asymptotically *χ*^2 ^with *D*(*q*) degrees of freedom, and in particular,

*E*(*δ*(q1^
 MathType@MTEF@5@5@+=feaafiart1ev1aaatCvAUfKttLearuWrP9MDH5MBPbIqV92AaeXatLxBI9gBaebbnrfifHhDYfgasaacH8akY=wiFfYdH8Gipec8Eeeu0xXdbba9frFj0=OqFfea0dXdd9vqai=hGuQ8kuc9pgc9s8qqaq=dirpe0xb9q8qiLsFr0=vr0=vr0dc8meaabaqaciaacaGaaeqabaqabeGadaaakeaadaqiaaqaaiabdghaXnaaBaaaleaacqaIXaqmaeqaaaGccaGLcmaaaaa@2FFF@, q2^
 MathType@MTEF@5@5@+=feaafiart1ev1aaatCvAUfKttLearuWrP9MDH5MBPbIqV92AaeXatLxBI9gBaebbnrfifHhDYfgasaacH8akY=wiFfYdH8Gipec8Eeeu0xXdbba9frFj0=OqFfea0dXdd9vqai=hGuQ8kuc9pgc9s8qqaq=dirpe0xb9q8qiLsFr0=vr0=vr0dc8meaabaqaciaacaGaaeqabaqabeGadaaakeaadaqiaaqaaiabdghaXnaaBaaaleaacqaIYaGmaeqaaaGccaGLcmaaaaa@3001@)) ~ 2*D*(*q*)/ℓ.

Using this result, one can perform *χ*^2 ^tests of equality between the C and V regions of EIAV. This yields p-values very close to zero. The biggest p-value is obtained for the two variable regions V1 and V2 and is 4·10^-17^. Since the p-values are so small, one can conclude that the Markov chains previously defined to model the C and V regions of EIAV do not reflect the same statistical composition in words of amino acids. Hence, each of the 9 constant regions and the 8 variable regions has a specific signature.

## Discussion

We report that HMMs are able to predict the C/V segmentations of various lentiviruses, based only on their deduced amino-acid sequences or their nucleotide sequences, with an amazing accuracy and a great robustness.

We would like to stress the fact that our algorithms identify the V regions without any comparison by alignment with known sequences. The models developed in this study are not based on computations of divergences between sequences. Furthermore, the lengths of the regions exhibit great variability, and the numbers of regions themselves may be, and indeed are sometimes, different from one sequence to another. These, and the various tests presented in section Results, prove that the models do not rely on the relative positions of the regions, nor on their lengths, to identify C/V segmentations of the sequences. On the contrary, they have to rely only on some statistical differences between the compositions in words of nucleotides or amino-acids of length 1 + *m*, where *m *is the order of the model.

More detailed consequences of the performances of the models are as follows. First, all the C regions can be suitably modeled by a unique state. This proves that they have similar statistical properties. The V regions can be modeled either by one state or by several states. This suggests that V regions share common properties, when compared to C regions, and, at the same time, that each V region has its own statistical profile.

**Figure 6:**
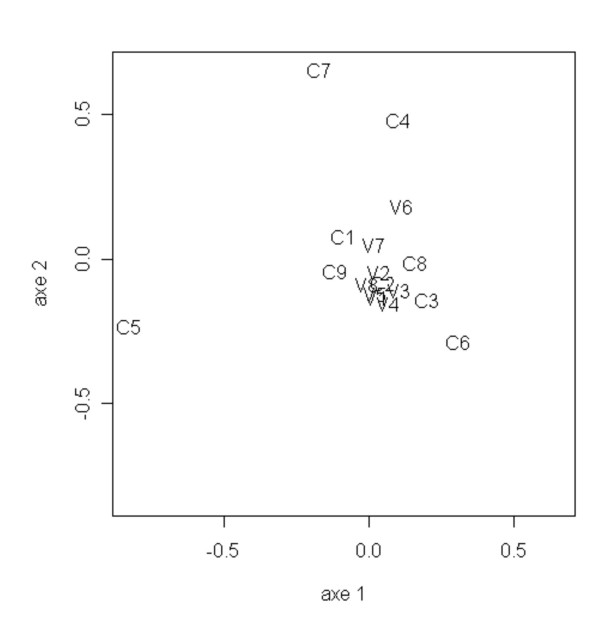
**Principal Components Analysis of the C/V regions of EIAV**. Plot of the two first axes of the principal components analysis of the composition in words of two amino-acids of the constant C1 to C9 and variable V1 to V8 regions of EIAV.

To highlight similarities and differences between data, a classical statistical method is based on Principal Components Analysis (PCA). Knowing that first order HMMs were able to differentiate between the C and V regions on deduced amino-acid sequences of EIAV SU and used only frequencies of words of two amino-acids, we performed a PCA of the 9 constant regions and the 8 variable regions of EIAV, using the frequencies of 20 × 20 = 400 words of two amino-acids as variables. Figure 6 shows a projection of the C and V regions of EIAV onto the plane defined by the two first principal axes. One sees that, contrary to our method based on HMMs, PCA does not allow to separate the EIAV regions into two groups, whether these groups correspond to the C regions and the V regions or not. With PCA, all the regions seem to have nearly the same statistical composition in words of two amino-acids, although it is not the case. Thus our method, based on HMMs, is able to reveal rather subtle differences between the group of V regions and the group of C regions.

It may be of interest to note that a model, trained on EIAV sequences only, failed to identify the C and V regions of other lentiviruses, and conversely. Hence, the genetic compositions of the *env *genes of these different lentiviruses are distinct. However, the C and V regions of EIAV, HIV, SIV and SRLV do share some properties which are similar enough, so as to be recognized by a unique HMM, trained on a combined pool of EIAV, HIV, SIV and SRLV SU sequences. This combined model also predicts the C/V segmentation of BIV and FIV, whose sequences were not used to train the model. This supports the conclusion that the statistical compositions in words of nucleotides or amino-acids of the envelope genes of all these lentiviruses share some common features.

Models of order *m *= 5 on nucleotide sequences, based on the frequencies of words of length 6, predict with an amazing accuracy the C/V segmentations. These words correspond to one or two complete codons. This length is also compatible with the number of nucleotides that are in the neighborhood of the palm of RT during the retrotranscription [27-29]. This suggests that some mechanism of inaccurate nucleotide substitution, possibly due to sequence-specific variations and in interaction with the side chains of the RT protein, might modify the speed and/or the precision of the passage along the portion of the RNA chain which the RT copies.

## Conclusion

The constant and variable regions of the lentiviruses EIAV, HIV, SLRV, SIV, BIV, and FIV can be identified by rather crude mathematical models based on HMMs. We attempt at present to extract the nature of the statistical signals which allow to distinguish between these regions. In this spirit, it has been reported that the retroviral G → A hypermutation occurs mainly in specific dinucleotide contexts, like GpG and GpA [30,31]. Hence, one of our objectives now is to compare to known contexts of mutation the nucleotide words which are, as the present study shows, statistically characteristic of the variable regions of these lentiviruses.

The most interesting contribution of the combined models based on nucleotide or deduced amino-acid sequences of different lentiviruses is the rapid identification of the C and V regions on newly identified lentivirus sequences without the requirement to alignment. This should be especially useful for highly divergent sequences.

HMMs are usually used to identify homogeneous segments in long sequences. In this study, we showed that HMMs are powerful tools able to identify very short segments in sequences of just few hundred nucleotides. We are considering to generalize our method to study small motifs in short sequences.

## Methods

### Biological data

This section describes the sets of SU nucleotide sequences, used to train and to test the models (Table 3). When the set of sequences for a virus was sufficiently large, like for EIAV or HIV, we chose to break down it into two equal parts and to use half of the sequences to train the models and half to test the models. When the number of available sequences of a virus is more limited, we chose to use 3/4 of the sequences for the traning set in order to have enougth data to estimate the parameters of the models.

**Table 3:** GenBank accession numbers of the sequences used in this study.

EIAV	AF005104 to AF005151 (except AF005113, AF005136 and AF005145 to AF005148); AF016316; AF298666 to AF298762 (except AF298752 and AF298691 to AF298694); AF429316 to AF429353
HIV	K03455; AB032740, AB03274; AF133821; AF190127, AF190128; AF197340; AF209205, AF209208; AF219261, AF219272; AF322202 to AF322214; AF411964, AF411965; AF413978, AF413979; AF413987; AF443113 to AF443115; AF457079 to AF457090 (except AF457082 to AF457084, AF457086 and AF457089); AF460972, AF460974; AF484478, AF484493; AF484507 à AF484519 (except AF484508, AF484510, AF484512 and AF484517); AF529572, AF529573; AF530576; AF544007, AF544008; AJ417424 to AJ417431; AY037268 to AY037270; AY037280 to AY037283; AY158533 to AY158535; AY173957, AY173958; AY217545; AY228556, AY228557; AY253305 to AY253322 (except AY253307, AY253309, AY253315 to AY253316 and AY253319); AY255823 to AY255827; AY322184 to AY322191 (except AY322186 and AY322188); AY357571 to AY357576 (except AY357574); AY358069 to AY358073 (except AY358070); AY371155 to AY371163 (except AY371158 to AY371162); AY423908 to AY423928; AY494965 to AY494974 (except AY494967 to AY494968, AY494970 and AY494972); AY505010, AY505011; AY535509 to AY535513; AY563169; AY818641 to AY818643

SIV	AF075269; AF103818; AF131870; AF188114 to AF188116; AF328295; AF334679; AF382828, AF382829; AF447763; AY033233; AY159321, AY159322; AY169968; AY221508 to AY221513; AY290709 to AY290716; AY523865 to AY523867; AY587015; AY588946; AY599198 to AY599201; AY611488; L20008, L20009; L20098, L20099; L40990; M29975; M33262; M58410; M66437; M83293; U04005; U10897 to U10898; U25712 to U25715; U25744, U25745; U58991; U72748

SRLV	A15114; AF015180; AF156858 to AF156877; AF338227; AF474005 to AF474007; AF479638; AJ400718 to AJ400721; AY039765 to AY039784; L06906; M31646; M33677; M34193; M60609, M60610; M60855; S51392; S55323; U35795 to U35804 (except U35797, U35802 and U35803); U51910

BIV	L43126 to L43132; M32690; NC_001413; L04972; U80989 to U80991

FIV	M25381; M36968; L00608; M59418; X57001 to X57002; M73964 to M73965; X60725; L06725; X69494 to X69502 (except X69495, X69500 and X69501)

• EIAV: 187 sequences [6,9,32-35].

Training set: 94 sequences. Test set: 93 sequences.

According to the regions described in [6], we considered 8 variable regions (V1 to V8) and 9 constant regions (C1 to C9).

• HIV: 155 HIV-1 sequences. The panel is composed of the HIV-1 HXB2 sequence and representative sequences from the following subtypes: A (21 sequences), B (27 sequences), C (26 sequences), D (18 sequences), E (19 sequences), F (3 sequences), G (21 sequences), H (2 sequences), and 17 sequences of recombinant forms.

Training set: 78 sequences. Test set: 77 sequences.

Variable regions V1 to V5 are as defined in [7]. However, V1 and V2 are considered as a unique variable region V1/V2, since these variable regions are separated by a small constant region composed of only a few nucleotides.

• SIV: 61 sequences. Training set: 45 sequences. Test set: 15 sequences.

Variable regions V1 to V5 are as defined in [5].

• SRLV: 68 sequences. Training set: 51 sequences. Test set: 17 sequences.

Variable regions V1 to V5 are as defined in [8].

• BIV: 13 sequences. Test set: 13 sequences.

We compared the predicted regions with the variable regions V1 to V6 previously defined in [10].

• FIV: 16 sequences. Test set: 16 sequences.

We compared the predicted regions with the variable regions V1 to V5 previously defined in [11].

### Hidden Markov models

We recalled in the introduction that HMMs involve pairs of random processes, called respectively the hidden process and the observed process. In our context, the hidden process (*S*_*i*_)_1 ≤ *i *≤ *L *_describes the succession of homogeneous regions along a sequence of length *L*. For every *i*, *S*_*i *_belongs to a given finite set of size *N *and is called the hidden state at position *i*. The observed process (*X*_*i*_)_1 ≤ *i *≤ *L *_describes the nucleotide sequence or the deduced amino-acid sequence. For every *i*, *X*_*i *_belongs to a given finite alphabet X
 MathType@MTEF@5@5@+=feaafiart1ev1aaatCvAUfKttLearuWrP9MDH5MBPbIqV92AaeXatLxBI9gBamrtHrhAL1wy0L2yHvtyaeHbnfgDOvwBHrxAJfwnaebbnrfifHhDYfgasaacH8akY=wiFfYdH8Gipec8Eeeu0xXdbba9frFj0=OqFfea0dXdd9vqai=hGuQ8kuc9pgc9s8qqaq=dirpe0xb9q8qiLsFr0=vr0=vr0dc8meaabaqaciaacaGaaeqabaWaaeGaeaaakeaaimaacqWFxepwaaa@384F@ of size *M *and is called the observation at position *i*. For instance, *M *:= 4 and X
 MathType@MTEF@5@5@+=feaafiart1ev1aaatCvAUfKttLearuWrP9MDH5MBPbIqV92AaeXatLxBI9gBamrtHrhAL1wy0L2yHvtyaeHbnfgDOvwBHrxAJfwnaebbnrfifHhDYfgasaacH8akY=wiFfYdH8Gipec8Eeeu0xXdbba9frFj0=OqFfea0dXdd9vqai=hGuQ8kuc9pgc9s8qqaq=dirpe0xb9q8qiLsFr0=vr0=vr0dc8meaabaqaciaacaGaaeqabaWaaeGaeaaakeaaimaacqWFxepwaaa@384F@ : = {A, C, G, T} for nucleotide sequences, and *M *:= 20 for deduced amino-acid sequences.

We use HMMs of type M1M*m*, hence the hidden process is a first order Markov chain. That is, the value *S*_*i *_at position *i *depends probabilistically on the value *S*_*i *- 1 _at position *i *- 1. The transition matrix *T *is defined as

*T*(*s'*|*s*) := *P*(*S*_*i *_= *s'*|*S*_*i*-1 _= *s*),

for every states *s *and *s'*, that is, *T*(*s'*|*s*) denotes the probability that *S*_*i *_= *s'*, conditionally on the fact that *S*_*i *- 1 _= *s*. In turn, conditionally on the state process, the observed process is an inhomogeneous Markov chain of order *m *whose transition probabilities at position *i *depend only on *S*_*i*_. The emission matrix *B *is defined as

*B*(*x *|*s*,*x*_1_, ..., *x*_*m*_) := *P*(*X*_*i *_= *x *| *X*_*i *- 1 _= *x*_1_,..., *X*_*i*-*m *_= *x*_*m*_, *S*_*i *_= *s*),

for every state *s *and every observations *x*, *x*_1_, ..., *x*_*m*_. In words, the state at a given position depends on the state at the previous position, and the observation at a given position depends on the state at the same position and on the *m *previous observations.

Hence, the full model is specified by the pair of matrices (*T*, *B*) and by some initial distributions.

### Parameter estimation

The estimation of the best model (*T*, *B*) for a given training set of sequences is usually based on maximum likelihood methods. Assume first that the segmentation of the observed sequences is available, that is, that one knows the state sequences. Then, the parameters of the model can be estimated with direct counting methods. For every states *s *and *s'*, one sets

T^(s′|s):=N(ss′)N(s),
 MathType@MTEF@5@5@+=feaafiart1ev1aaatCvAUfKttLearuWrP9MDH5MBPbIqV92AaeXatLxBI9gBaebbnrfifHhDYfgasaacH8akY=wiFfYdH8Gipec8Eeeu0xXdbba9frFj0=OqFfea0dXdd9vqai=hGuQ8kuc9pgc9s8qqaq=dirpe0xb9q8qiLsFr0=vr0=vr0dc8meaabaqaciaacaGaaeqabaqabeGadaaakeaacuWGubavgaqcaiabcIcaOiqbdohaZzaafaGaeiiFaWNaem4CamNaeiykaKIaeiOoaOJaeyypa0ZaaSaaaeaacqWGobGtcqGGOaakcqWGZbWCcuWGZbWCgaqbaiabcMcaPaqaaiabd6eaojabcIcaOiabdohaZjabcMcaPaaacqGGSaalaaa@4102@

where *N*(*s*), respectively *N*(*ss'*), denotes the number of times the letter *s*, respectively the word *ss'*, appears in the state sequence, that is,

N(s):=∑i=1L1{Si=s},N(ss′):=∑i=1L−11{Si=s,Si+1=s′}.
 MathType@MTEF@5@5@+=feaafiart1ev1aaatCvAUfKttLearuWrP9MDH5MBPbIqV92AaeXatLxBI9gBaebbnrfifHhDYfgasaacH8akY=wiFfYdH8Gipec8Eeeu0xXdbba9frFj0=OqFfea0dXdd9vqai=hGuQ8kuc9pgc9s8qqaq=dirpe0xb9q8qiLsFr0=vr0=vr0dc8meaabaqaciaacaGaaeqabaqabeGadaaakeaafaqabeqacaaabaGaemOta4KaeiikaGIaem4CamNaeiykaKIaeiOoaOJaeyypa0ZaaabCaeaaieqacqWFXaqmcqGG7bWEcqWGtbWudaWgaaWcbaGaemyAaKgabeaakiabg2da9iabdohaZjabc2ha9jabcYcaSaWcbaGaemyAaKMaeyypa0JaeGymaedabaGaemitaWeaniabggHiLdaakeaacqWGobGtcqGGOaakcqWGZbWCcuWGZbWCgaqbaiabcMcaPiabcQda6iabg2da9maaqahabaGae8xmaeJaei4EaSNaem4uam1aaSbaaSqaaiabdMgaPbqabaGccqGH9aqpcqWGZbWCcqGGSaalcqWGtbWudaWgaaWcbaGaemyAaKMaey4kaSIaeGymaedabeaakiabg2da9iqbdohaZzaafaGaeiyFa0NaeiOla4caleaacqWGPbqAcqGH9aqpcqaIXaqmaeaacqWGmbatcqGHsislcqaIXaqma0GaeyyeIuoaaaaaaa@660C@

Likewise, for every observations *x*, *x*_1_, ..., *x*_*m*_, and every state *s*, one sets

B^(x|s,x1,...,xm):=N(xm⋯x1x|s)N(xm⋯x1∗|s),
 MathType@MTEF@5@5@+=feaafiart1ev1aaatCvAUfKttLearuWrP9MDH5MBPbIqV92AaeXatLxBI9gBaebbnrfifHhDYfgasaacH8akY=wiFfYdH8Gipec8Eeeu0xXdbba9frFj0=OqFfea0dXdd9vqai=hGuQ8kuc9pgc9s8qqaq=dirpe0xb9q8qiLsFr0=vr0=vr0dc8meaabaqaciaacaGaaeqabaqabeGadaaakeaacuWGcbGqgaqcaiabcIcaOiabdIha4jabcYha8jabdohaZjabcYcaSiabdIha4naaBaaaleaacqaIXaqmaeqaaOGaeiilaWIaeiOla4IaeiOla4IaeiOla4IaeiilaWIaemiEaG3aaSbaaSqaaiabd2gaTbqabaGccqGGPaqkcqGG6aGocqGH9aqpdaWcaaqaaiabd6eaojabcIcaOiabdIha4naaBaaaleaacqWGTbqBaeqaaOGaeS47IWKaemiEaG3aaSbaaSqaaiabigdaXaqabaGccqWG4baEcqGG8baFcqWGZbWCcqGGPaqkaeaacqWGobGtcqGGOaakcqWG4baEdaWgaaWcbaGaemyBa0gabeaakiabl+UimjabdIha4naaBaaaleaacqaIXaqmaeqaaOGaey4fIOIaeiiFaWNaem4CamNaeiykaKcaaiabcYcaSaaa@5F04@

with

N(xm⋯x1∗|s):=∑z∈XN(xm⋯x1z|s).
 MathType@MTEF@5@5@+=feaafiart1ev1aaatCvAUfKttLearuWrP9MDH5MBPbIqV92AaeXatLxBI9gBaebbnrfifHhDYfgasaacH8akY=wiFfYdH8Gipec8Eeeu0xXdbba9frFj0=OqFfea0dXdd9vqai=hGuQ8kuc9pgc9s8qqaq=dirpe0xb9q8qiLsFr0=vr0=vr0dc8meaabaqaciaacaGaaeqabaqabeGadaaakeaacqWGobGtcqGGOaakcqWG4baEdaWgaaWcbaGaemyBa0gabeaakiabl+UimjabdIha4naaBaaaleaacqaIXaqmaeqaaOGaey4fIOIaeiiFaWNaem4CamNaeiykaKIaeiOoaOJaeyypa0ZaaabuaeaacqWGobGtcqGGOaakcqWG4baEdaWgaaWcbaGaemyBa0gabeaakiabl+UimjabdIha4naaBaaaleaacqaIXaqmaeqaaOGaemOEaONaeiiFaWNaem4CamNaeiykaKcaleaacqWG6bGEcqGHiiIZt0uy0HwzTfgDPnwy1egaryqtHrhAL1wy0L2yHvdaiqaacqWFxepwaeqaniabggHiLdGccqGGUaGlaaa@5D98@

Here *N*(*x*_*m *_... *x*_1_*z*|*s*) is the number of times the word *x*_*m *_... *x*_1_*z *appears in the observation sequence while the state is *s *at the position of the observation *x*, that is,

N(xm⋯x1z|s):=∑i=m+1L1{Xi−m=xi−m,Xi−1=xi−1,Xi=z,Si=s}.
 MathType@MTEF@5@5@+=feaafiart1ev1aaatCvAUfKttLearuWrP9MDH5MBPbIqV92AaeXatLxBI9gBaebbnrfifHhDYfgasaacH8akY=wiFfYdH8Gipec8Eeeu0xXdbba9frFj0=OqFfea0dXdd9vqai=hGuQ8kuc9pgc9s8qqaq=dirpe0xb9q8qiLsFr0=vr0=vr0dc8meaabaqaciaacaGaaeqabaqabeGadaaakeaacqWGobGtcqGGOaakcqWG4baEdaWgaaWcbaGaemyBa0gabeaakiabl+UimjabdIha4naaBaaaleaacqaIXaqmaeqaaOGaemOEaONaeiiFaWNaem4CamNaeiykaKIaeiOoaOJaeyypa0ZaaabCaeaaieqacqWFXaqmcqGG7bWEcqWGybawdaWgaaWcbaGaemyAaKMaeyOeI0IaemyBa0gabeaakiabg2da9iabdIha4naaBaaaleaacqWGPbqAcqGHsislcqWGTbqBaeqaaOGaeiilaWIaemiwaG1aaSbaaSqaaiabdMgaPjabgkHiTiabigdaXaqabaGccqGH9aqpcqWG4baEdaWgaaWcbaGaemyAaKMaeyOeI0IaeGymaedabeaakiabcYcaSiabdIfaynaaBaaaleaacqWGPbqAaeqaaOGaeyypa0JaemOEaONaeiilaWIaem4uam1aaSbaaSqaaiabdMgaPbqabaGccqGH9aqpcqWGZbWCcqGG9bqFcqGGUaGlaSqaaiabdMgaPjabg2da9iabd2gaTjabgUcaRiabigdaXaqaaiabdYeambqdcqGHris5aaaa@6E8E@

As is well known, maximum likelihood estimators are sensitive to overfitting. To avoid such problems, we added constant pseudo-counts *n*_0 _to every *N*(*s*), *N*(*ss'*) and *N*(*x*_*m *_... *x*_1_*z*|*s*), equal to *n*_0 _:= 1.

### Reconstruction algorithms

When the segmentation of the training sequences is not available, the maximum likelihood estimators (T^
 MathType@MTEF@5@5@+=feaafiart1ev1aaatCvAUfKttLearuWrP9MDH5MBPbIqV92AaeXatLxBI9gBaebbnrfifHhDYfgasaacH8akY=wiFfYdH8Gipec8Eeeu0xXdbba9frFj0=OqFfea0dXdd9vqai=hGuQ8kuc9pgc9s8qqaq=dirpe0xb9q8qiLsFr0=vr0=vr0dc8meaabaqaciaacaGaaeqabaqabeGadaaakeaacuWGubavgaqcaaaa@2DED@, B^
 MathType@MTEF@5@5@+=feaafiart1ev1aaatCvAUfKttLearuWrP9MDH5MBPbIqV92AaeXatLxBI9gBaebbnrfifHhDYfgasaacH8akY=wiFfYdH8Gipec8Eeeu0xXdbba9frFj0=OqFfea0dXdd9vqai=hGuQ8kuc9pgc9s8qqaq=dirpe0xb9q8qiLsFr0=vr0=vr0dc8meaabaqaciaacaGaaeqabaqabeGadaaakeaacuWGcbGqgaqcaaaa@2DC9@) of (*T*, *B*) cannot be directly computed. But there exists several algorithms which estimate iteratively the parameters of the models with no foreknowledge of either the observation process or the state process. The most classical one is the expectation-maximization (EM) algorithm, introduced by [36]. In the context of hidden Markov chains, this algorithm is known as the Baum-Welch algorithm, see [13,37] for a detailed description of the algorithm, and [12]. To compute maximum likelihood estimates of the parameters, this algorithm alternates E-steps and M-steps until convergence. During each E-step, the algorithm estimates the missing data (the hidden states sequence), computing the most likely state sequence with respect to the current value of the parameters, obtained through the preceding M-step. During each M-step, the algorithm maximizes the transition and emission probabilities, using the state sequence computed during the preceding E-step. There is no guarantee that the EM algorithm should produce a sequence (*T*_*n*_, *B*_*n*_)_*n *≥ 0 _of models which converges to (T^
 MathType@MTEF@5@5@+=feaafiart1ev1aaatCvAUfKttLearuWrP9MDH5MBPbIqV92AaeXatLxBI9gBaebbnrfifHhDYfgasaacH8akY=wiFfYdH8Gipec8Eeeu0xXdbba9frFj0=OqFfea0dXdd9vqai=hGuQ8kuc9pgc9s8qqaq=dirpe0xb9q8qiLsFr0=vr0=vr0dc8meaabaqaciaacaGaaeqabaqabeGadaaakeaacuWGubavgaqcaaaa@2DED@, B^
 MathType@MTEF@5@5@+=feaafiart1ev1aaatCvAUfKttLearuWrP9MDH5MBPbIqV92AaeXatLxBI9gBaebbnrfifHhDYfgasaacH8akY=wiFfYdH8Gipec8Eeeu0xXdbba9frFj0=OqFfea0dXdd9vqai=hGuQ8kuc9pgc9s8qqaq=dirpe0xb9q8qiLsFr0=vr0=vr0dc8meaabaqaciaacaGaaeqabaqabeGadaaakeaacuWGcbGqgaqcaaaa@2DC9@). Indeed, starting from an unspecified initial point (*T*_0_, *B*_0_), the algorithm can get stuck in one of many local maxima of the likelihood. But there exists a neighborhood of (T^
 MathType@MTEF@5@5@+=feaafiart1ev1aaatCvAUfKttLearuWrP9MDH5MBPbIqV92AaeXatLxBI9gBaebbnrfifHhDYfgasaacH8akY=wiFfYdH8Gipec8Eeeu0xXdbba9frFj0=OqFfea0dXdd9vqai=hGuQ8kuc9pgc9s8qqaq=dirpe0xb9q8qiLsFr0=vr0=vr0dc8meaabaqaciaacaGaaeqabaqabeGadaaakeaacuWGubavgaqcaaaa@2DED@, B^
 MathType@MTEF@5@5@+=feaafiart1ev1aaatCvAUfKttLearuWrP9MDH5MBPbIqV92AaeXatLxBI9gBaebbnrfifHhDYfgasaacH8akY=wiFfYdH8Gipec8Eeeu0xXdbba9frFj0=OqFfea0dXdd9vqai=hGuQ8kuc9pgc9s8qqaq=dirpe0xb9q8qiLsFr0=vr0=vr0dc8meaabaqaciaacaGaaeqabaqabeGadaaakeaacuWGcbGqgaqcaaaa@2DC9@), such that, for any (*T*_0_, *B*_0_) in this neighborhood, (*T*_*n*_, *B*_*n*_)_*n *≥ 0 _indeed converges to (T^
 MathType@MTEF@5@5@+=feaafiart1ev1aaatCvAUfKttLearuWrP9MDH5MBPbIqV92AaeXatLxBI9gBaebbnrfifHhDYfgasaacH8akY=wiFfYdH8Gipec8Eeeu0xXdbba9frFj0=OqFfea0dXdd9vqai=hGuQ8kuc9pgc9s8qqaq=dirpe0xb9q8qiLsFr0=vr0=vr0dc8meaabaqaciaacaGaaeqabaqabeGadaaakeaacuWGubavgaqcaaaa@2DED@, B^
 MathType@MTEF@5@5@+=feaafiart1ev1aaatCvAUfKttLearuWrP9MDH5MBPbIqV92AaeXatLxBI9gBaebbnrfifHhDYfgasaacH8akY=wiFfYdH8Gipec8Eeeu0xXdbba9frFj0=OqFfea0dXdd9vqai=hGuQ8kuc9pgc9s8qqaq=dirpe0xb9q8qiLsFr0=vr0=vr0dc8meaabaqaciaacaGaaeqabaqabeGadaaakeaacuWGcbGqgaqcaaaa@2DC9@) ([38,39]). To introduce some information about the composition of the different regions, we also define and use a new algorithm based on the EM algorithm and on direct counting methods. The details of this new algorithm are as follows. The emission matrix *B*, corresponding to the transition probabilities between observations for each state, is defined by counting on training sequences. Then one estimates iteratively the state transition probabilities of the *T *matrix with the EM algorithm, keeping every emission probabilities at their calculated value. The M-step of the EM algorithm is modified, to omit the usual maximization of the emission probabilities. Then, the E-step and the maximization of the transition probabilities are performed as in the classical EM algorithm. We call this new algorithm fixed EM algorithm (which stands for EM algorithm with fixed emission probabilities). In details, the fixed EM algorithm produces a sequence (*T*_*n*_, *B*_*n*_)_*n *≥ 0 _of models as follows.

#### Step Initiation

The transition probabilities *T*_0 _are initialized using random values. The emission matrix *B*_0 _is defined by counting on training sequences as follows:

B0(x|s,x1,...,xm):=N(xm⋯x1x|s)N(xm⋯x1∗|s),
 MathType@MTEF@5@5@+=feaafiart1ev1aaatCvAUfKttLearuWrP9MDH5MBPbIqV92AaeXatLxBI9gBaebbnrfifHhDYfgasaacH8akY=wiFfYdH8Gipec8Eeeu0xXdbba9frFj0=OqFfea0dXdd9vqai=hGuQ8kuc9pgc9s8qqaq=dirpe0xb9q8qiLsFr0=vr0=vr0dc8meaabaqaciaacaGaaeqabaqabeGadaaakeaacqWGcbGqdaWgaaWcbaGaeGimaadabeaakiabcIcaOiabdIha4jabcYha8jabdohaZjabcYcaSiabdIha4naaBaaaleaacqaIXaqmaeqaaOGaeiilaWIaeiOla4IaeiOla4IaeiOla4IaeiilaWIaemiEaG3aaSbaaSqaaiabd2gaTbqabaGccqGGPaqkcqGG6aGocqGH9aqpdaWcaaqaaiabd6eaojabcIcaOiabdIha4naaBaaaleaacqWGTbqBaeqaaOGaeS47IWKaemiEaG3aaSbaaSqaaiabigdaXaqabaGccqWG4baEcqGG8baFcqWGZbWCcqGGPaqkaeaacqWGobGtcqGGOaakcqWG4baEdaWgaaWcbaGaemyBa0gabeaakiabl+UimjabdIha4naaBaaaleaacqaIXaqmaeqaaOGaey4fIOIaeiiFaWNaem4CamNaeiykaKcaaiabcYcaSaaa@6018@

with the same notations than in the section "Parameter estimation".

#### Step Estimation (E)

Computation of the probability *P*_*k*, ℓ _of every successives states *k *and ℓ in *S*, under the current value (*T*_*n*_, *B*_*n*_).

*P*_*k*,ℓ _= *P*(*S*_*i *_= *k*, *S*_*i*+1 _= ℓ|*x*_1_, ..., *x*_*L*_, (*T*_*n*_, *B*_*n*_)).

This probability can be computed using the forward and backward variables *f*_*k*_(*i*) and *b*_*k*_(*i*) defined by:

*f*_*k*_(*i*) = *P*(*x*_1_, ..., *x*_*i*_, *S*_*i *_= *k*),

and

*b*_*k*_(*i*) = *P*(*x*_*i*+1_, ..., *x*_*n *_| *S*_*i *_= *k*, *x*_*i*-*m*+1_, ..., *x*_*i*_).

We have:

Pk,ℓ=fk(i)⋅Tn(k,ℓ)⋅Bn(ℓ,xi−m+1:i,xi+1)⋅bℓ(i+1)∑k∈Sfk(n),
 MathType@MTEF@5@5@+=feaafiart1ev1aaatCvAUfKttLearuWrP9MDH5MBPbIqV92AaeXatLxBI9gBaebbnrfifHhDYfgasaacH8akY=wiFfYdH8Gipec8Eeeu0xXdbba9frFj0=OqFfea0dXdd9vqai=hGuQ8kuc9pgc9s8qqaq=dirpe0xb9q8qiLsFr0=vr0=vr0dc8meaabaqaciaacaGaaeqabaqabeGadaaakeaacqWGqbaudaWgaaWcbaGaem4AaSMaeiilaWIaeS4eHWgabeaakiabg2da9maalaaabaGaemOzay2aaSbaaSqaaiabdUgaRbqabaGccqGGOaakcqWGPbqAcqGGPaqkcqGHflY1cqWGubavdaWgaaWcbaGaemOBa4gabeaakiabcIcaOiabdUgaRjabcYcaSiabloriSjabcMcaPiabgwSixlabdkeacnaaBaaaleaacqWGUbGBaeqaaOGaeiikaGIaeS4eHWMaeiilaWIaemiEaG3aaSbaaSqaaiabdMgaPjabgkHiTiabd2gaTjabgUcaRiabigdaXiabcQda6iabdMgaPjabcYcaSaqabaGccqWG4baEdaWgaaWcbaGaemyAaKMaey4kaSIaeGymaedabeaakiabcMcaPiabgwSixlabdkgaInaaBaaaleaacqWItecBaeqaaOGaeiikaGIaemyAaKMaey4kaSIaeGymaeJaeiykaKcabaWaaabeaeaacqWGMbGzdaWgaaWcbaGaem4AaSgabeaaaeaacqWGRbWAcqGHiiIZt0uy0HwzTfgDPnwy1egaryqtHrhAL1wy0L2yHvdaiqaacqWFse=uaeqaniabggHiLdGccqGGOaakcqWGUbGBcqGGPaqkaaGaeiilaWcaaa@7BC5@

#### Step Maximization (M)

Computation of (*T*_*n*+1_, *B*_*n*+1_), through the formulas

Tn+1(k,ℓ)=t(k,ℓ)∑s∈St(k,s),
 MathType@MTEF@5@5@+=feaafiart1ev1aaatCvAUfKttLearuWrP9MDH5MBPbIqV92AaeXatLxBI9gBaebbnrfifHhDYfgasaacH8akY=wiFfYdH8Gipec8Eeeu0xXdbba9frFj0=OqFfea0dXdd9vqai=hGuQ8kuc9pgc9s8qqaq=dirpe0xb9q8qiLsFr0=vr0=vr0dc8meaabaqaciaacaGaaeqabaqabeGadaaakeaacqWGubavdaWgaaWcbaGaemOBa4Maey4kaSIaeGymaedabeaakiabcIcaOiabdUgaRjabcYcaSiabloriSjabcMcaPiabg2da9maalaaabaGaemiDaqNaeiikaGIaem4AaSMaeiilaWIaeS4eHWMaeiykaKcabaWaaabeaeaacqWG0baDcqGGOaakcqWGRbWAcqGGSaalcqWGZbWCcqGGPaqkaSqaaiabdohaZjabgIGioprtHrhAL1wy0L2yHvtyaeHbnfgDOvwBHrxAJfwnaGabaiab=jr8tbqab0GaeyyeIuoaaaGccqGGSaalaaa@563E@

where

t(k,ℓ)=∑i=1n−1fk(i)⋅Tn(k,ℓ)⋅Bn(ℓ,xi−m+1:i,xi+1)⋅bℓ(i+1),
 MathType@MTEF@5@5@+=feaafiart1ev1aaatCvAUfKttLearuWrP9MDH5MBPbIqV92AaeXatLxBI9gBaebbnrfifHhDYfgasaacH8akY=wiFfYdH8Gipec8Eeeu0xXdbba9frFj0=OqFfea0dXdd9vqai=hGuQ8kuc9pgc9s8qqaq=dirpe0xb9q8qiLsFr0=vr0=vr0dc8meaabaqaciaacaGaaeqabaqabeGadaaakeaacqWG0baDcqGGOaakcqWGRbWAcqGGSaalcqWItecBcqGGPaqkcqGH9aqpdaaeWbqaaiabdAgaMnaaBaaaleaacqWGRbWAaeqaaOGaeiikaGIaemyAaKMaeiykaKIaeyyXICTaemivaq1aaSbaaSqaaiabd6gaUbqabaGccqGGOaakcqWGRbWAcqGGSaalcqWItecBcqGGPaqkcqGHflY1cqWGcbGqdaWgaaWcbaGaemOBa4gabeaakiabcIcaOiabloriSjabcYcaSiabdIha4naaBaaaleaacqWGPbqAcqGHsislcqWGTbqBcqGHRaWkcqaIXaqmcqGG6aGocqWGPbqAcqGGSaalaeqaaOGaemiEaG3aaSbaaSqaaiabdMgaPjabgUcaRiabigdaXaqabaGccqGGPaqkcqGHflY1cqWGIbGydaWgaaWcbaGaeS4eHWgabeaakiabcIcaOiabdMgaPjabgUcaRiabigdaXiabcMcaPiabcYcaSaWcbaGaemyAaKMaeyypa0JaeGymaedabaGaemOBa4MaeyOeI0IaeGymaedaniabggHiLdaaaa@6FFA@

and

*B*_*n*+1 _= *B*_0_.

#### Step End

The steps E and M are executed alternatively until convergence.

The fixed EM algorithm converges to the maximum likelihood estimators (T˜
 MathType@MTEF@5@5@+=feaafiart1ev1aaatCvAUfKttLearuWrP9MDH5MBPbIqV92AaeXatLxBI9gBaebbnrfifHhDYfgasaacH8akY=wiFfYdH8Gipec8Eeeu0xXdbba9frFj0=OqFfea0dXdd9vqai=hGuQ8kuc9pgc9s8qqaq=dirpe0xb9q8qiLsFr0=vr0=vr0dc8meaabaqaciaacaGaaeqabaqabeGadaaakeaacuWGubavgaacaaaa@2DEC@, B˜
 MathType@MTEF@5@5@+=feaafiart1ev1aaatCvAUfKttLearuWrP9MDH5MBPbIqV92AaeXatLxBI9gBaebbnrfifHhDYfgasaacH8akY=wiFfYdH8Gipec8Eeeu0xXdbba9frFj0=OqFfea0dXdd9vqai=hGuQ8kuc9pgc9s8qqaq=dirpe0xb9q8qiLsFr0=vr0=vr0dc8meaabaqaciaacaGaaeqabaqabeGadaaakeaacuWGcbGqgaacaaaa@2DC8@) conditioned by the emission matrix *B*. The model (T˜
 MathType@MTEF@5@5@+=feaafiart1ev1aaatCvAUfKttLearuWrP9MDH5MBPbIqV92AaeXatLxBI9gBaebbnrfifHhDYfgasaacH8akY=wiFfYdH8Gipec8Eeeu0xXdbba9frFj0=OqFfea0dXdd9vqai=hGuQ8kuc9pgc9s8qqaq=dirpe0xb9q8qiLsFr0=vr0=vr0dc8meaabaqaciaacaGaaeqabaqabeGadaaakeaacuWGubavgaacaaaa@2DEC@, B˜
 MathType@MTEF@5@5@+=feaafiart1ev1aaatCvAUfKttLearuWrP9MDH5MBPbIqV92AaeXatLxBI9gBaebbnrfifHhDYfgasaacH8akY=wiFfYdH8Gipec8Eeeu0xXdbba9frFj0=OqFfea0dXdd9vqai=hGuQ8kuc9pgc9s8qqaq=dirpe0xb9q8qiLsFr0=vr0=vr0dc8meaabaqaciaacaGaaeqabaqabeGadaaakeaacuWGcbGqgaacaaaa@2DC8@) yields a lower likelihood than the model (T^
 MathType@MTEF@5@5@+=feaafiart1ev1aaatCvAUfKttLearuWrP9MDH5MBPbIqV92AaeXatLxBI9gBaebbnrfifHhDYfgasaacH8akY=wiFfYdH8Gipec8Eeeu0xXdbba9frFj0=OqFfea0dXdd9vqai=hGuQ8kuc9pgc9s8qqaq=dirpe0xb9q8qiLsFr0=vr0=vr0dc8meaabaqaciaacaGaaeqabaqabeGadaaakeaacuWGubavgaqcaaaa@2DED@, B^
 MathType@MTEF@5@5@+=feaafiart1ev1aaatCvAUfKttLearuWrP9MDH5MBPbIqV92AaeXatLxBI9gBaebbnrfifHhDYfgasaacH8akY=wiFfYdH8Gipec8Eeeu0xXdbba9frFj0=OqFfea0dXdd9vqai=hGuQ8kuc9pgc9s8qqaq=dirpe0xb9q8qiLsFr0=vr0=vr0dc8meaabaqaciaacaGaaeqabaqabeGadaaakeaacuWGcbGqgaqcaaaa@2DC9@) obtained with the EM algorithm. Experimentally, on EIAV sequences, we observe that the convergence of fixed EM occurs 10 times faster than the convergence of the EM algorithm. We defined the fixed EM algorithm to introduce some information about the number *N *of types of regions and the statistical composition in words of nucleotides or amino-acids of these regions. On the contrary, we introduced no information about the order or the position of the regions along the sequence.

In both EM and fixed EM algorithms, to reconstruct the hidden states sequence and to identify the predicted C and V regions, one determines the sequence of the most probable hidden states, that is, one computes at each position *i *of the sequence the likelihood of the different hidden states (*S*_*i *_= *s*) conditionally on the observed sequence and one selects the state with the highest likelihood. The likelihood of the hidden states for each position is computed using the classical forward-backward algorithm, described by [40].

## Authors' contributions

ABQ developed the methods, performed the *in silico *experiments and drafted the manuscript under the supervision of CL and DP. DP conceived the statistical test described in the appendix. CL and DP conceived, designed and coordinated the project, assured its supervision, participated in the interpretation of data and helped to draft the manuscript. All authors read and approved the final manuscript.

## Supplementary Material

Additional file 1 On the discrimination of Markov chains through their empirical transition matrices. The Additional file 1 describes an asymptotic statistical test to discriminate Markov chains through their empirical transition matrices. Click here for file
