# Supplementary material for: In silico segmentations of lentivirus envelope sequences
Source: BMC Bioinformatics. 2007 Mar 21;8:99. doi: 10.1186/1471-2105-8-99 (PMC1847453; doi:10.1186/1471-2105-8-99)
Supplement: Additional file 1 — On the discrimination of Markov chains through their empirical transition matrices. The Additional file 1 describes an asymptotic statistical test to discriminate Markov chains through their empirical transition matrices. [file 1471-2105-8-99-S1.pdf]

Appendix: On the discrimination of Markov chains through their empirical transition matrices

Aurélia Boissin-Quillon[1], Didier Piau[2] and Caroline Leroux[1]

[1] UMR754 INRA-ENVL-UCBL"Rétrovirus et Pathologie Comparée", IFR 128 BioSciences Lyon-Gerland, Université Claude Bernard Lyon 1, Domaine de Gerland, 69007 Lyon, France

[2] Institut Fourier UMR 5582 CNRS-UJF, Université Joseph Fourier (Grenoble 1), 100 rue des Maths, BP 74, 38402 Saint Martin d'Hères, France

Email: Aurélia Boissin-Quillon - aurelia.quillon@univ-lyon1.fr; Didier Piau - Didier.Piau@ujf-grenoble.fr; Caroline Leroux*- caroline.leroux@univ-lyon1.fr;

*Corresponding author

Introduction

We consider irreducible Markov chains on a finite number k of states, with transition matrix q and stationary distribution p. The number ℓ of edges used by the chain is the number of couples of states (x, y) such that $q(x, y) > 0$, hence $k \leq \ell \leq k^2$. When $\ell = k$, the chain moves deterministically on an oriented discrete circle, hence one can exclude this case if necessary. On the contrary, as soon as $\ell \geq k + 1$, several trajectories are possible and the chain is truly random. Finally, $\ell = k^2$ means that all the transitions are allowed, hence the chain moves on the complete graph with loops. The dimension $D(q)$ of the chain is

$$D(q) := \ell - k,$$

This the number of free parameters among the nonzero coefficients of q, that is, the dimension of the simplex formed by the transition matrices subordinated to q, in the sense that the coefficients corresponding to coefficients of q equal to 0 have to be equal to 0 too.

The maximum likelihood estimator $\widehat{q}$ of q uses countings along a trajectory of length n and is a consistent estimator of q when n goes to infinity. The relation

$$\widehat{q}(x, y) = q(x, y) + z_{xy}/\sqrt{n} + o(1/\sqrt{n}),$$

defines a Gaussian centered vector $(z_{xy})_{xy}$, indexed by the edges (x, y), and whose covariance matrix is an explicit function of p and q.

We consider the relative entropy of the empirical measure, given by the observed trajectory, with respect to the theoretical measure, given by p and q. This random entropy is defined by

$$H(\widehat{q},q) := \sum_{(x,y)} \widehat{p}_x \, \widehat{q}(x,y) \, \log(\widehat{q}(x,y)/q(x,y)),$$

where the sum indexed by (x,y) has ℓ terms and $\widehat{p}$ denotes the stationary distribution of $\widehat{q}$. One can also consider the entropy

$$H(q,\widehat{q}) := \sum_{(x,y)} p_x \, q(x,y) \, \log(q(x,y)/\widehat{q}(x,y)).$$

Using second-order Taylor series approximations of the logarithm function, one sees that both $H(\widehat{q},q)$ and $H(q,\widehat{q})$ are such that, when n becomes large,

$$H = h/(2n) + o(1/n), \quad h := \sum_{(x,y)} z_{xy}^2 \, p_x/q(x,y).$$

In this appendix we show that the reduced relative entropy h follows a quite simple χ^2 distribution and we draw some statistical consequences from this result.

Convergence in distribution

Let N_x, respectively N_{xy}, denote the number of times the vertice x, respectively the edge (x,y), is visited up to time n. Consider

$$\xi_{xy} := (N_{xy} - q(x,y)N_x)/\sqrt{N_x}.$$

According to [P. Billingsley (1960). Statistical Inference in Markov Chain. The Stanford meetings of the Institute of Mathematical Statistics. Statistical Research Monographs, Vol. II. The University of Chicago Press, Chicago, Ill. 1961], the matrices $(\xi_{xy})_{xy}$ converge in distribution, when n goes to infinity, to a Gaussian centered matrix $(g_{xy})_{xy}$ distributed as follows. The vectors $(g_{xy})_y$ are independent for different states x, hence the covariance of g_{xy} and g_{zt} is 0 for every $x \neq z$ and every y and t. Finally, for every x, y and z,

$$E(g_{xy}g_{xz}) = -q(x,y)q(x,z) \quad (y \neq z), \qquad E(g_{xy}^2) = q(x,y)(1 - q(x,y)).$$

Since N_x/n converges almost surely to p_x, one can replace the factor $1/\sqrt{N_x}$ by $1/\sqrt{np_x}$. This remark yields the following convergence in distribution:

$$np_x(\widehat{q}(x,y) - q(x,y))^2 \to g_{xy}^2.$$

In addition, we recall that, if one observes an i.i.d. sequence with theoretical distribution p on k states, then the empirical distribution $\widehat{p}$ is such that $2nH(\widehat{p}, p)$ converges in distribution to a χ^2 distribution with $k-1$ degrees of freedom.

The vectors $(g_{xy})_{xy}$ are independent. Furthermore, for each fixed x, $(g_{xy})_y$ admits the same covariances that the limit gaussian distribution obtained for an i.i.d. sequence of distribution $q(x, \cdot)$. In addition, the random variable

$$H_x := \sum_y q(x,y)\log(q(x,y)/\widehat{q}(x,y))$$

corresponds to the observation of this i.i.d. process during a time which corresponds to the number of visits of x before n, that is, a random number of visits which is $np_x + o(n)$. Hence, $2(np_x)H_x$ converges in distribution to the χ^2 distribution with $D_x(q)$ degrees of freedom, where $D_x(q)+1$ equal the number of y such that $q(x,y) > 0$. By independance of the limits in distribution of the $2np_xH_x$, their sum $2nH$ converges in distribution to the χ^2 distribution with $D(q)$ degrees of freedom, where $D(q)$ is the sum indexed by x of the $D_x(q)$.

In conclusion, h follows the χ^2 distribution with $D(q)$ degrees of freedom.

Statistical applications

Assume that one has two independent sequences of observations of the same Markov chain with transition matrix q. This yields two estimators $\widehat{q}_1$ and $\widehat{q}_2$ of q, based respectively on the countings $N^{(1)}$ and $N^{(2)}$. We proved the relations

$$\widehat{q}_i(x,y) = q(x,y) + z^{(i)}_{xy}/\sqrt{n} + o(1/\sqrt{n}), \quad i = 1, 2,$$

where the two families $(z^{(1)}_{xy})_{xy}$ and $(z^{(2)}_{xy})_{xy}$ are independent and follow the distribution of $(z_{xy})_{xy}$ described in the previous section. The reduced relative entropy between the two sequences of observations is asymptotically equal to

$$h(\widehat{q}_1, \widehat{q}_2) := \sum_{(x,y)} (z^{(1)}_{xy} - z^{(2)}_{xy})^2 \alpha_{xy},$$

where α_{xy} can be indifferently $p^{(1)}_x/q_1(x,y)$ or $p^{(2)}_x/q_2(x,y)$ or $p_x/q(x,y)$. If one uses $\alpha_{xy} = p_x/q(x,y)$, then $h(\widehat{q}_1, \widehat{q}_2)$ follows exactly the distribution of $2h(\widehat{q}, q)$, and the same result holds asymptotically for the other choices of $\alpha_{x,y}$. Hence, to determine whether $\widehat{q}_1$ and $\widehat{q}_2$ correspond to the same Markov chain or not, one can use the fact that, if they do, $nH(\widehat{q}_1, \widehat{q}_2)$ is asymptotically χ^2 with $D(q)$ degrees of freedom. In particular,

$$E(H(\widehat{q}_1, \widehat{q}_2)) \sim D(q)/n.$$

If one wishes to work instead with the symmetrized form of the relative entropy, one can use

$$\zeta := n(H(\widehat{q}_1, \widehat{q}_2) + H(\widehat{q}_2, \widehat{q}_1)) = \sum_{(x,y)} (N^{(1)}_{xy} - N^{(2)}_{xy}) \log \left(\frac{N^{(1)}_{xy} N^{(2)}_x}{N^{(1)}_x N^{(2)}_{xy}} \right).$$

Since ζ is asymptotically twice a χ^2 with $D(q)$ degrees of freedom, one can compute the p-value of the event $\{\zeta \geq t\}$ for every $t \geq 2D(q)$.

To compute upper bounds of the p-values of χ^2 distributions of large dimension d, one can use exponential Cramer bounds. This yields that, for every $t \geq d$, the probability that a χ^2 distribution with d degrees of freedom is greater than t is at most

$$e^{-t/2}(te/d)^{d/2}.$$

This approximation yields for instance that, if $d = 400 - 20 = 380$, the p-value for $t = 460$ is less than 2.47 % and the p-value for $t = 480$ is less than 0.04 %, to be compared to the true p-values 0.3% and 0.03% respectively.
